# *In-situ* Monitoring of Internal Local Temperature and Voltage of Proton Exchange Membrane Fuel Cells

**DOI:** 10.3390/s100706395

**Published:** 2010-06-29

**Authors:** Chi-Yuan Lee, Wei-Yuan Fan, Wei-Jung Hsieh

**Affiliations:** Department of Mechanical Engineering, Yuan Ze Fuel Cell Center, Yuan Ze University, Taoyuan, Taiwan; E-Mails: s975040@mail.yzu.edu.tw (W.-Y.F.); s965034@mail.yzu.edu.tw (W.-J.H.)

**Keywords:** flexible multi-functional micro sensors, MEMS, PEMFC

## Abstract

The distribution of temperature and voltage of a fuel cell are key factors that influence performance. Conventional sensors are normally large, and are also useful only for making external measurements of fuel cells. Centimeter-scale sensors for making invasive measurements are frequently unable to accurately measure the interior changes of a fuel cell. This work focuses mainly on fabricating flexible multi-functional microsensors (for temperature and voltage) to measure variations in the local temperature and voltage of proton exchange membrane fuel cells (PEMFC) that are based on micro-electro-mechanical systems (MEMS). The power density at 0.5 V without a sensor is 450 mW/cm^2^, and that with a sensor is 426 mW/cm^2^. Since the reaction area of a fuel cell with a sensor is approximately 12% smaller than that without a sensor, but the performance of the former is only 5% worse.

## 1. Introduction

Global energy requirements are increasing daily. The threat of global warming due to the burning of fossil fuels has focused attention on the environment and the need for efficient and clean energy sources. Fuel cells have attracted considerable attention due to their advantages of high efficiency, low noise, low pollution, low fuel selectivity, and wide ranging potential applications.

In the future, fuel cells will become an important source of electrical power, but for this to happen some problems must still be resolved. For instance, determining the temperature and humidity within a fuel cell is extremely difficult. Numerous investigations have identified the important factors associated with the effects of cell temperature, fuel temperature, as well as other factors governing cell performance [1,2]. A thermistor can be a thin film. It can be used to measure directly the temperature in membrane electrode assembly (MEA). Sensors reduce cell performance by 20% although the physical processes in the cells may not be significantly changed [1].

There are various non-invasive methods for taking measurements to reveal water and thermal conditions inside fuel cells, however, these normally require large, complex and costly instruments such as infrared or neutron beams [3–5]. In most studies, a small temperature sensor is inserted into fuel cells. For example, David employed fiber Bragg grating technology to research the temperature distribution in fuel cells [6]. Liu, Hwang and others have adopted separated plates to export electric power and analyze it to measure fuel cell performance [7,8]. Sun, Zhang *et al.* exported and analyzed electrical energy by inserting a metal wire into a fuel cell [9,10].

However, in the cited investigations, the bipolar plates were cut, and mm to cm–scale sensors were inserted into fuel cells, not only increasing the contact resistance, and increasing the possibility of fuel leakage, but also changing the cell environment and making the measurements inaccurate. Although some researchers have increased the resolution by miniaturization and thus reduced the effect on the performance of the fuel cells, enabling comparison of average performance associated with several membrane, gas diffusion layer (GDL) materials and Pt loadings [11,12].

The references indicate that sensors degrade fuel cell performance, mostly by enhancing contact resistance, blocking the path of gas diffusion, or directly blocking the path of proton transfer. In the authors’ other work, micro-flexible temperature and humidity sensors were successfully fabricated on a parylene substrate [13,14]. However, these sensors had the (drawbacks or shortcomings) of: (1) being unuseable in high-temperature environments (>200 °C); (2) not supporting the use of a wire-bonder to make interconnection lines between the lines of the sensor pad.

Therefore, in this investigation, stainless steel foil (40 μm-thick) was used in the fabrication process as a flexible substrate to overcome the abovementioned issues. Stainless steel foil has a high corrosion resistance, high compression resistance, high temperature resistance and high flexibility. This work presents a novel approach for the *in-situ* monitoring of internal local temperature and voltage of proton exchange membrane fuel cells using flexible multi-functional temperature and voltage microsensors, which were fabricated using micro-electro-mechanical systems (MEMS) technology. The flexible multi-functional microsensors have the advantages of: (1) small size, (2) high sensitivity, (3) flexible but precise measurement positions, and (4) *in-situ* measurement.

## 2. Methodology

**Figure 1.**
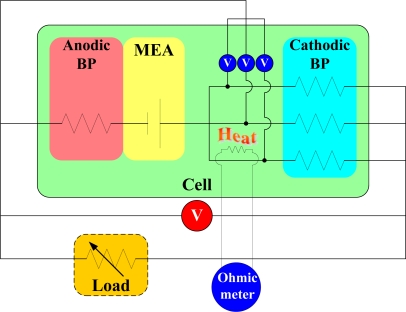
Schematic of the temperature and voltage measurement system.

In this investigation, the temperature sensor was a resistance temperature detector (RTD). As the environmental temperature increases, the resistance of the RTD also increases, because a metal conductor has a positive temperature coefficient (PTC). When the temperature of the RTD varies linearly, the relationship between the measured resistance and the change in temperature can be expressed as:

(1) $$R_{t}=R_{i}\left(1+\alpha_{T}T\right)$$

where *R_t_* is the resistance at *t* °C; *R_i_* is the resistance at *i* °C, and α*_T_* is the sensitivity (1/°C) [15]. This temperature sensor was used in the fuel cell. Figure 1 shows the temperature and voltage measurement system.

## 3. Fabrication of Flexible Multi-functional Micro Sensors

**Figure 2.**
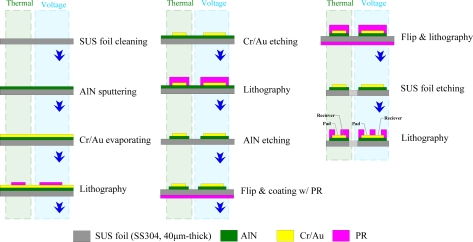
Flowchart for fabricating micro temperature and voltage sensors.

**Figure 3.**
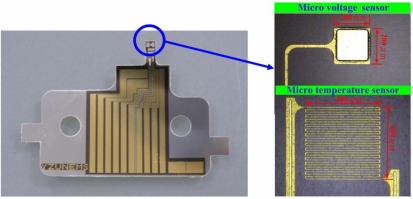
Optical microscopic photograph of micro temperature and voltage sensors.

In this study flexible multi-functional microsensors (temperature and voltage) were fabricated to measure the local temperature and voltage variations of a proton exchange membrane fuel cell (PEMFC) using micro-electro-mechanical systems (MEMS) techniques. Figure 2 presents the steps in the fabrication of a flexible micro temperature and voltage sensor:

First, sulfuric acid and hydrogen peroxide were used to clean the stainless steel foil (40 μm). Aluminum nitride (AlN, 1 μm) was sputtered as a bottom insulation layer. An E-beam evaporator was then applied to evaporate chromium (Cr, 400 Å) as an adhesive layer between AlN and gold (Au, (2,000 Å), and evaporated gold was used to form the micro temperature and voltage sensors by wet etching. Finally, aluminum nitride (0.5 μm) was sputtered as a top insulation layer, and the micro temperature and voltage sensors were connected using an Al wire. Figure 3 presents flexible multi-functional microsensors, comprising micro temperature and voltage sensors, with areas of 400 μm × 400 μm and 200 μm × 200 μm, respectively.

## 4. Results and Discussion

**Figure 4.**
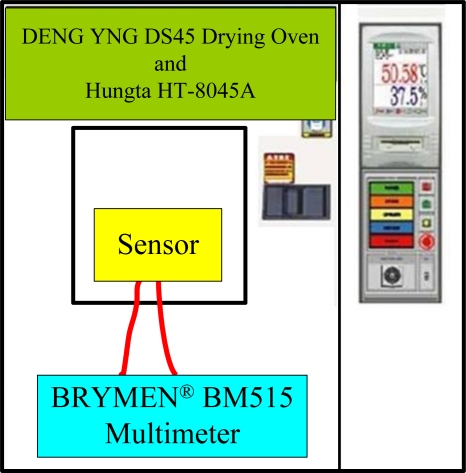
Programmable temperature calibration system.

**Figure 5.**
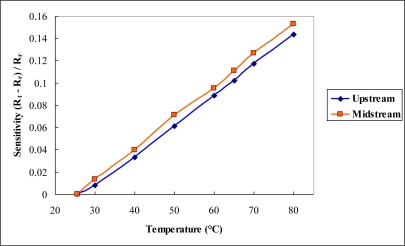
Calibration curves of micro temperature sensors in upstream and midstream.

**Figure 6.**
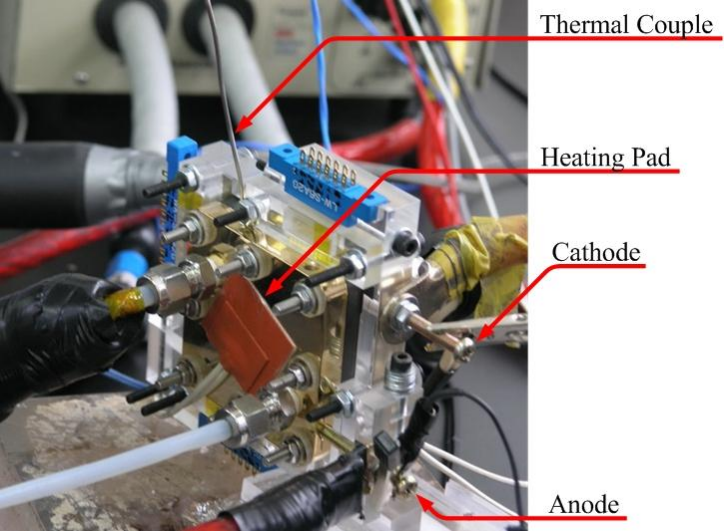
Fuel cell testing system.

**Figure 7.**
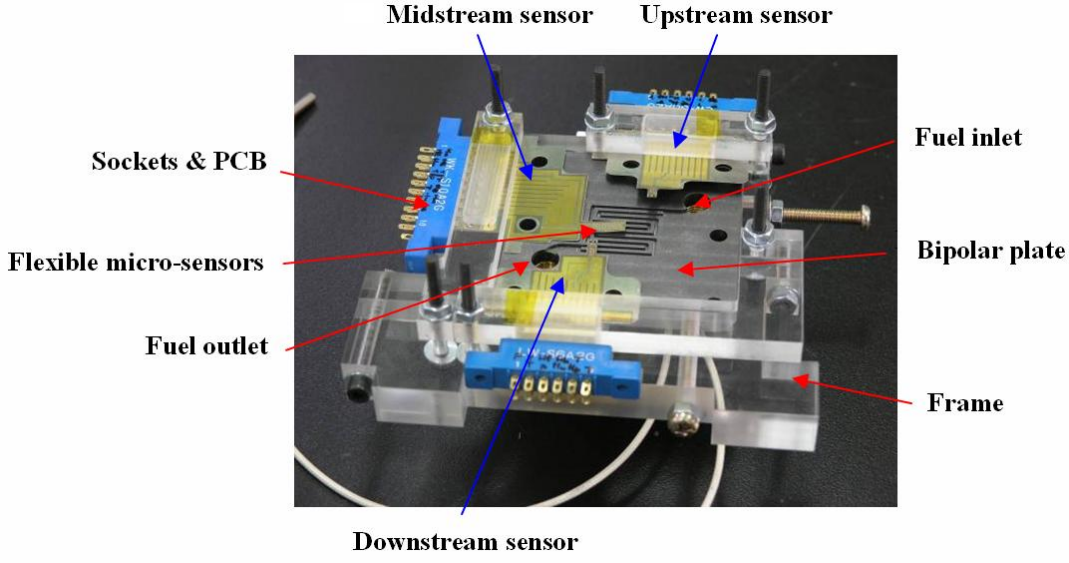
*In-situ* diagnostic device embedded in a PEM fuel cell.

**Figure 8.**
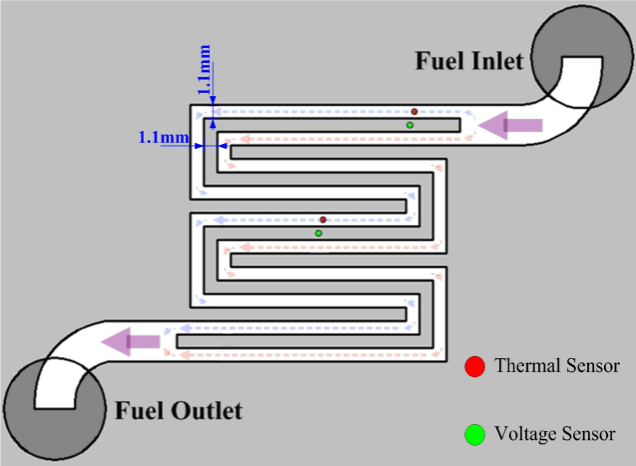
Locations of micro sensors.

After the flexible multi-functional micro sensors (temperature and voltage) have been formed, they were calibrated using a programmable temperature chamber, as shown in Figures 4. Figure 5 shows the calibration curves for the micro temperature sensors upstream and midstream. Figure 6 presents the fuel cell testing system. Figure 7 displays the *in-situ* diagnostic device in the PEM fuel cell. Figure 8 shows the locations of the microsensors.

In this work, the cell temperature was 65 °C, and the relative humidity was 100%. The anode channel supplies H_2_ at flow rate of 120 SCCM, and the cathode channel supplies O_2_ at a flow rate of 365 SCCM. The membrane electrode assembly (MEA) was E-TEK ES12E-W-5L-12E-W. The endplate was brass and the bipolar plate was graphite. The reaction area was 5.29 cm^2^. Table 1 presents the other conditions and specifications of the flow channel. Table 2 presents the fuel cell testing flowchart, continuing to constant voltage, constant circuit, and constant power, respectively.

### 4.1. Constant Voltage Test

**Figure 9.**
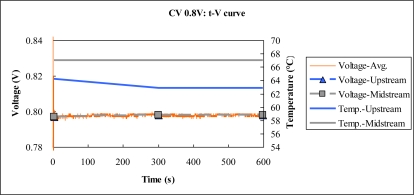
Voltage and temperature difference at constant voltage 0.8 V.

**Figure 10.**
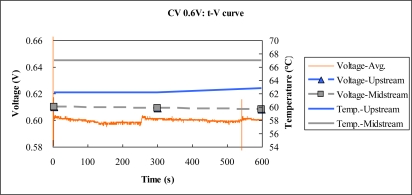
Voltage and temperature difference at constant voltage 0.6 V.

**Figure 11.**
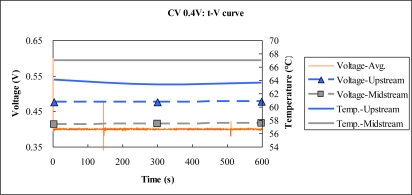
Voltage and temperature difference at constant voltage 0.4 V.

Figures 9 to 11 plot the output temperature and potential at constant voltages 0.8 V, 0.6 V and 0.4 V. The figures indicate that the temperature difference between upstream and midstream is approximately 3 °C. The temperature upstream becomes more different from the midstream one with time, while the midstream temperature is quite stable.

### 4.2. Constant Current Test

**Figure 12.**
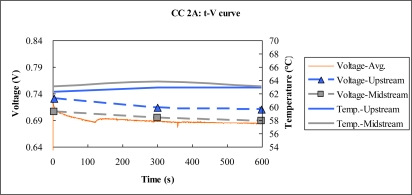
Voltage and temperature difference at 2 A constant current.

**Figure 13.**
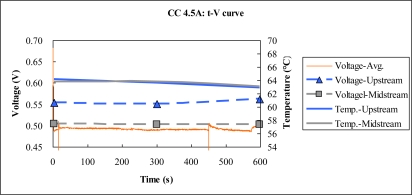
Voltage and temperature difference at 4.5 A constant current.

Figures 12 to 13 plot the output temperature and voltage at constant currents of 2 A and 4.5 A. According to these figures, the temperature difference between upstream and midstream is around 1 °C. At the higher current output, the difference between the power densities upstream and midstream is about 5.86 mW/cm^2^.

### 4.3. Constant P Test

**Figure 14.**
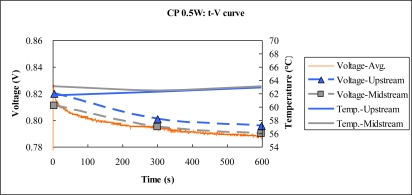
Voltage and temperature difference at constant power 0.5 W.

**Figure 15.**
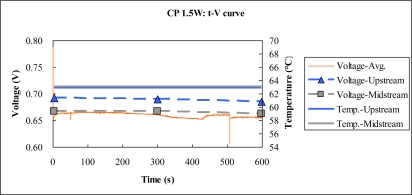
Voltage and temperature difference at constant power 1.5 W.

Figures 14 to 15 plot the output temperature and voltage at constant powers of 1.5 W and 0.5 W. The power density at constant power 1.5 W exceeds that at constant power 0.5 W, consistent with the two aforementioned results.

### 4.4. Comparison with Polarization Curve

**Figure 16.**
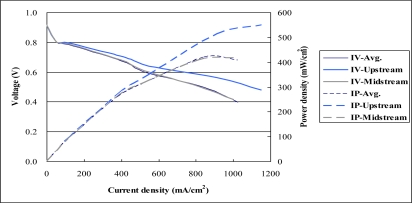
Comparison of cell performance with and without sensors.

Figure 16 plots polarization curves upstream, midstream and for the whole fuel cell. The midstream performance is very close to that of the whole fuel cell. The power density at 0.5 V without sensor is 450 mW/cm^2^, and that with the sensor is 426 mW/cm^2^. Since the reaction area of the fuel cell with the sensor is approximately 12% less than that without the sensor, the difference in performance is around 5%. The performance degrades due to enhanced contact resistance and the fact that the masked area of the microsensors blocks the proton transfer path. Table 3 shows the comparison of power density with and without the sensor. The upstream performance is better than that of the whole fuel cell, because the right amount of fuel is available upstream, while flooding occur midstream.

## 5. Conclusions

This study has successfully integrated micro temperature and voltage sensors in a stainless steel foil with a thickness of 40 μm. The sensors are used to determine real time variations in the local temperature and voltage of a proton exchange membrane fuel cell. Experimental results indicate that the value of the potential inner fuel cell exceeds the outer measured value due to the difference in contact resistance. Additionally, the performance of the upstream is better than that midstream in a fuel cell, because a sufficient amount of fuel is upstream, where less flooding occurs that midstream. Future improvements are warranted to modulate the testing flowchart in order to confirm whether the experimental results are the same.

## Figures and Tables

**Table 1.** Operating conditions and specifications of flow-channel.

**Items**	**Conditions**
Cell temperature	65 °C
Relative humidity (%RH)	100 %
H_2_ flow rate (Anode)	120 SCCM (λ = 3x @ 1A/cm^2^)
Air flow rate (Cathode)	365 SCCM (λ = 3.8x @ 1A/cm^2^)
MEA	E-TEK
Bipolar plate/Flow field type	Graphite/Dual-path serpentine
Flow-channel depth	1.1 mm
Flow-channel width	1.1 mm
Flow-rib width	1.1 mm
Reaction area	5.29 cm^2^

**Table 2.** Fuel cell testing flowchart.

**Motion**	**Time**
Fuel cell heating to 65°C	About 30 minutes
Open circuit voltage testing (OCV)	Each 10 minutes
CV 0.8V	
CV 0.6V	
CV 0.4V	
CC 2.0A	
CC 4.5A	
CP 0.5W	
CP 1.5W	

**Table 3.** Comparison of power density with and without the sensor.

	**Power density at 0.5 V**	**Maximum power density**
Without microsensors	450 mW/cm^2^	463 mW/cm^2^
With microsensors	426 mW/cm^2^ (degrade 5.3 %)	420 mW/cm^2^ (degrade 9.3 %)

## References

[1] Mench MM, He S, Tadigadapa S (2006). Thin film temperature sensor for real-time measurement of electrolyte temperature in a polymer electrolyte fuel cell. Sens. Actuat. A.

[2] Lee CY, Wu GW, Hsieh CL (2007). In situ diagnosis of micrometallic proton exchange membrane fuel cells using microsensors. J. Power Sources.

[3] Wang M, Guo H, Ma C (2006). Temperature distribution on the MEA surface of a PEMFC with serpentine channel flow bed. J. Power Sources.

[4] Trabold TA, Owejan JP, Jacobson DL, Arif M, Huffman PR (2006). In situ investigation of water transport in an operating PEM fuel cell using neutron radiography: Part 1—Experimental method and serpentine flow field results. Int. J. Heat Mass Transfer.

[5] Owejan JP, Trabold TA, Jacobson DL, Baker DR, Hussey DS, Arif M (2006). In situ investigation of water transport in an operating PEM fuel cell using neutron radiography: Part 2—Transient water accumulation in an interdigitated cathode flow field. Int. J. Heat Mass Transfer.

[6] David NA, Wild PM, Hu J, Djilali N (2009). In-fibre Bragg grating sensors for distributed temperature measurement in a polymer electrolyte membrane fuel cell. J. Power Sources.

[7] Liu Z, Mao Z, Wu B, Wang L, Schmidt VM (2005). Current density distribution in PEFC. J. Power Sources.

[8] Hwang JJ, Chang WR, Peng RG, Chen PY, Su A (2008). Experimental and numerical studies of local current mapping on a PEM fuel cell. Int. J. Hydrogen Energy.

[9] Sun H, Zhang GL, Guo J, Liu H (2006). A novel technique for measuring current distributions in PEM fuel cells. J. Power Sources.

[10] Zhang G, Guo L, Ma B, Liu H (2009). Comparison of current distributions in proton exchange membrane fuel cells with interdigitated and serpentine flow fields. J. Power Sources.

[11] Kaytakoğlu S, Akyalçın L (2007). Optimization of parametric performance of a PEMFC. Int. J. Hydrogen Energy.

[12] Akyalçın L, Kaytakoğlu S (2008). Optimization of structural combinations on the performance of a PEMFC’s MEA. J. Power Sources.

[13] Lee CY, Wu GW, Hsieh WJ (2008). Fabrication of micro sensors on a flexible substrate. Sens. Actuat. A.

[14] Lee CY, Hsieh WJ, Wu GW (2008). Embedded flexible micro-sensors in MEA for measuring temperature and humidity in a micro-fuel cell. J. Power Sources.

[15] Wilson JS (2004). Sensor technology handbook.

